# A review of the biological effects of *Myrtus communis*

**DOI:** 10.14814/phy2.15770

**Published:** 2023-07-18

**Authors:** Mohammad Mahdi Dabbaghi, Mohammad Saleh Fadaei, Hesan Soleimani Roudi, Vafa Baradaran Rahimi, Vahid Reza Askari

**Affiliations:** ^1^ International UNESCO Center for Health‐Related Basic Sciences and Human Nutrition Mashhad University of Medical Sciences Mashhad Iran; ^2^ Department of Cardiovascular Diseases, Faculty of Medicine Mashhad University of Medical Sciences Mashhad Iran; ^3^ Applied Biomedical Research Center Mashhad University of Medical Sciences Mashhad Iran

**Keywords:** anti‐inflammatory, antioxidant, Myrtle, *Myrtus communis*, toxin

## Abstract

The World Health Organization stated that 1.6 million deaths worldwide were caused by contact with chemicals and toxins in 2019. In the same year, the Centers for Disease Control and Prevention stated that natural toxins caused 3960 deaths. *Myrtus communis*, also known as common Myrtle, is a flowering plant native to the Mediterranean region. Myrtle has been traditionally used to treat diarrhea, inflammation, bleeding, headache, pulmonary and skin diseases. This review was performed to assess Myrtle's protective and therapeutic efficacy against various chemical, natural, and radiational noxious. Multiple databases such as PubMed, Web of Sciences, and Scopus were investigated without publication time limitation. Recent studies have demonstrated its potential as a protective agent against both natural and chemical toxins. One of Myrtle's most significant protective properties is its high antioxidant content. Studies have shown that the antioxidant properties of Myrtle can protect against harmful substances such as heavy metals, pesticides, and other environmental toxins. Additionally, Myrtle has anti‐inflammatory properties that can help reduce the damage caused by long‐term exposure to toxins. The anti‐inflammatory and antimicrobial properties of Myrtle have also proven effective in alleviating gastrointestinal conditions such as gastric ulcers.

## 1 INTRODUCTION

Myrtaceae is a family of woody flowering plants with approximately 5500 species divided into 144 genera and 17 tribes. In this family, the tribe Myrteae includes half of the family's biodiversity, with 51 genera and approximately 2500 species found primarily in the Neotropics. *Myrtus* is the only genus found in Europe, North Africa, Asia, and especially in the Mediterranean region of southern Europe as far west as Macaronesia (Madeira and the Azores), the Saharan mountains, and as far east as western Asia (Hosseini et al., 2023; Migliore et al., 2012; Thornhill et al., 2015; Vasconcelos et al., 2017; Yahyazadeh et al., 2021).

**FIGURE 1:**
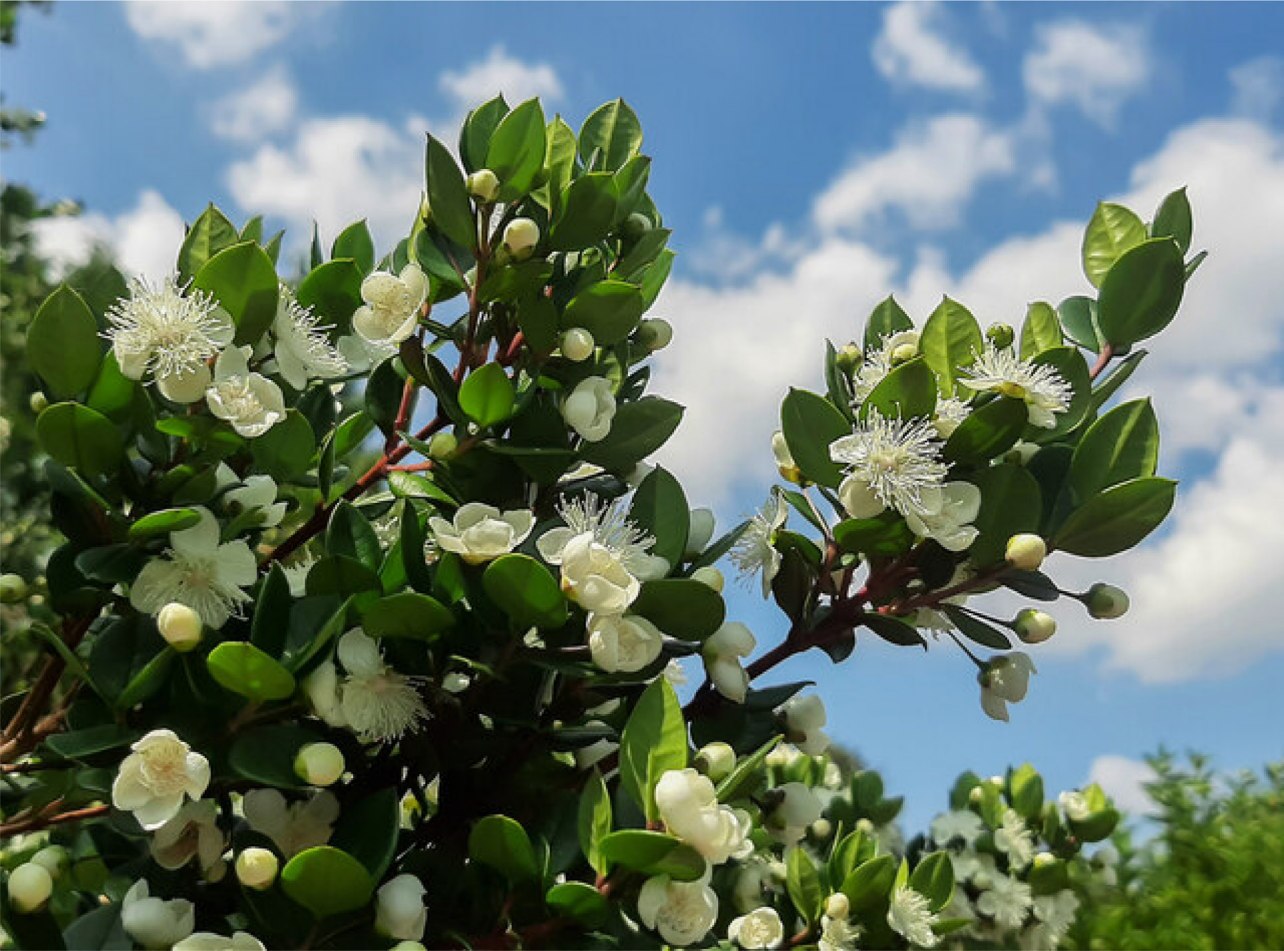
*Myrtus communis* (This photo was taken by Jonathan Billinger, near to Huntley, Gloucestershire, England, licensed for reuse under Creative Commons License; Attribution‐ShareAlike 4.0 International [CC BY‐SA 4.0]).

One of the most mentioned types of *Myrtus* in traditional books is *Myrtus communis*, also referred to as “Murt” or “Murd” (Mahboubi, 2017). The Mediterranean shrub *M. communis*, also known as common Myrtle, is native to the Mediterranean region. The plant is 2.4–3 m tall, with branches forming a close full head that is densely covered in leaves (Sumbul et al., 2011). Fruits are small and dark, with small green leaves (Asif et al., 2011). The evergreen leaves range in length from 2 to 5 cm. It has a bitter taste owing to its astringent properties (Alipour et al., 2014; Gortzi et al., 2008; Özkan & Güray, 2009). Flowers are star‐shaped, white or pinkish in color, and extremely fragrant (Charles, 2012). There are several seeds in the round blue‐black berry fruit. Insects pollinate the flowers, and birds that eat the berries spread the seeds (Figure 1) (Satyavati et al., 1987).

**FIGURE 2:**
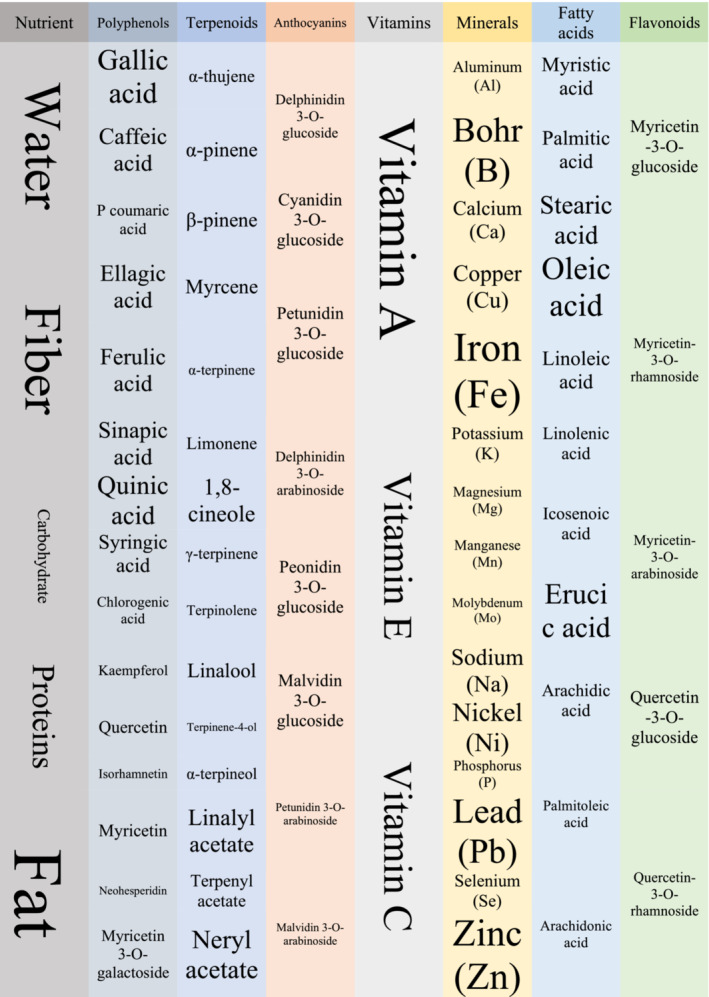
Active constituents of *Myrtus communis*.

It also has other active constituents like polyphenols, anthocyanins, and vitamins, which are shown in Figure 2 (Çakmak et al., 2021; Correddu et al., 2019; Hacıseferoğulları et al., 2012; Hennia et al., 2018; Messaoud & Boussaid, 2011; Ruffoni et al., 2009; Tuberoso et al., 2006). Because of its medicinal and aromatic effects, it is used for the treatment of patients in traditional medicine. Its medicinal uses are because of essential oils and compounds found in its leaves and fruit (Usai et al., 2018). The essential oil is extracted by hydro‐distillation from leaves and employed in the flavor and perfume industries. Myrtle berries and leaves are mostly used in the production of sweet liquor with digestive characteristics (Nuvoli & Spanu, 1996). Many studies have confirmed its antioxidant, antibacterial, and antifungal properties (Chalchat et al., 2010; Özcan & Akgül, 1995; Özcan & Boyraz, 2000; Özcan et al., 2015; Saǧdıç & Özcan, 2003). Myrtle essential oil has antimicrobial, insecticidal, antioxidant, and hepatoprotective properties (Hennia et al., 2019).

In traditional healers, Myrtle has been used to treat diarrhea, peptic ulcers, hemorrhoids, inflammation, uterine bleeding, headache, palpitation, leukorrhea, urethritis, epistaxis, conjunctivitis, excessive perspiration, pulmonary and skin diseases (Alipour et al., 2014; Mobli et al., 2015). In addition, because of their astringent, tonic, and antiseptic properties, Myrtle leaves have been used to treat wounds and digestive and urinary system disorders (Alipour et al., 2014; Sisay & Gashaw, 2017). Although berries decoctions were used for bathing babies with inflamed skin and leaves and berries decoctions in painful washing, the majority of the berries are utilized to manufacture the distinctive Myrtle liqueur derived by the hydroalcoholic extraction of the berries (Alipour et al., 2014; Fadda et al., 2017; Montoro et al., 2006).

We live in a world where our health is influenced by several elements, including climate, food, and toxins, via the multiple routes of administration. Toxic agents can have a variety of negative health impacts based on their physical, chemical, or biological characteristics, resulting in organ dysfunction. The seriousness of the harm is determined by exposure doses and durations, as well as individual factors such as age, diet, illness, pregnancy, and other relevant factors (Yahyazadeh et al., 2021). Toxins are biomolecules produced primarily for defensive purposes by bacteria, fungi, insects, plants, and vertebrate and invertebrate animals. These molecules cause harm to other organisms through inhalation, injection, ingestion, or absorption (Dorner & Rummel, 2015). The consequences of these toxins are frequently irreversible, resulting in permanent health harm. Toxins significantly impact health, food, and security (Janik et al., 2019).

In 2019, the World Health Organization (WHO) reported that 1.6 million deaths worldwide were caused by exposure to chemicals and toxins. In the same year, the Centers for Disease Control and Prevention reported that 3960 deaths were caused by natural toxins (Yahyazadeh et al., 2021).

This review will explain the treatment of different diseases, which are induced by various toxins, with Myrtle.

## 2 METHOD

A comprehensive search has been performed from Scopus, ScienceDirect, Web of Sciences, and PubMed databases without date limitation from inception to the first of April 2023. In this review article, all in vitro and in vivo studies were considered. In addition, the following medical keywords were investigated alone or in combinations: “embryotoxicity, genotoxicity, hematological toxicity, hepatotoxicity, ototoxicity, pulmonary toxicity, radiotoxicity, retinotoxicity, or skin phototoxicity” “toxic, toxicity, nephrotoxic, radiation, cardiotoxic, hepatotoxic, mycotoxins, pesticides, or cardiotoxicity” “neurotoxic, fumonisins, natural toxins, chemical toxicity, cardiotoxins, neurotoxins, nephrotoxins, ochratoxin, aflatoxins” “venoms, bacterial toxins, lipopolysaccharides (LPS), chemical agents induced toxicity, metals, cadmium, titanium dioxide, lead, or 6‐hydroxydopamine” “1‐methyl‐4‐phenylpyridinium (MPP), amyloid‐beta, dieldrin, chlorpyrifos, gentamicin, carbon tetrachloride, thioacetamide, diethyl nitrosamine, or azathioprine” “cisplatin, naphthalene, carbon tetrachloride, cardiotoxic agent, isoproterenol, ethanol, hydrogen peroxide, penicillic acid, or ergosterol” “radiation‐induced toxicity, ultraviolet, heavy metals, chromium, mercury, aluminum, doxorubicin, bleomycin, or diclofenac sodium” “thiourea, carbendazim, dextran sulfate sodium, carbon tetrachloride, phorbol, strychnine, 1‐methyl‐4‐phenyl‐1,2,3,6‐tetrahydropyridine (MTPT), streptozotocin (STZ)” “acetaminophen, hepatotoxins, or liver” with “*M. communis*.”

## 3 FINDINGS AND DISCUSSION

We have provided explanations about various chemical and natural toxins and the protective effect of Myrtle against them. The promising results of Myrtle have been reported in different in vitro and in vivo studies (Tables 1 and 2).

**TABLE 1:** In vivo protective effects of *Myrtus communis* against toxins and noxious agents.

Toxins/noxious	Models	Constituents	Results	References
Aflatoxin B1	Chicken	Myrtle essential oil (500 mg/kg) for 35 days	↓AST	(Saei et al., 2013)
			↓ALP	
			↓ALT	
Alcohol	Rats	Myrtle aqueous extract (105 and 175 mg/kg) and methanolic extract (93 and 154 mg/kg)	↓Gastric ulcer index	(Sumbul et al., 2010)
			↓Gastric juice volume	
			↑Gastric ph	
Aluminum chloride and D‐galactose	Rats	Myrtle extract (100–200 mg/kg) for 90 days	↓Aβ	(Yalman et al., 2022)
			↓8‐OHdG	
			↓Acetylcholinesterase activity	
			↑Neprilysin	
			↑SOD	
Arsenic	Rats	Myrtle leaf extract (3 mg/mL)	↑P53 gene expression	(Naji et al., 2018)
			↑PGE level	
Bleomycin	Rats	Myrtle leaf extract (50 mg/kg) for 14 days	↓Lipid peroxidation	(Samareh Fekri et al., 2018)
Carbon tetrachloride	Rats	Essential oil of Myrtle (250 mL/kg) for 14 days	↑LDH	(Ben Hsouna et al., 2019)
			↓TG	
			↑HDL‐Ch	
			↓T‐Ch	
			↑LDL‐Ch	
			↑Atherogenic index	
			↓TBARS	
			↓PCO	
			↑SOD	
			↑CAT	
			↑GPx	
Carrageenan	Mice	Myrtucommulone (0.5, 1.5, and 4.5 mg/kg) for 4 h	↓ICAM‐1	(Rossi et al., 2009)
			↓P‐selectin	
			↓TNF‐α	
			↓IL‐1β	
			↓Nitrotyrosine	
			↓PAR	
			↓MPO activity	
Castor oil	Rats	Myrtle berry seeds extract (25, 50, and 100 mg/kg)	↓MDA	(Jabri, Rtibi, et al., 2016)
			↑GSH	
			↑SOD	
			↑CAT	
			↑GPx	
			↓Wet fecal weight	
			↓Wet fecal no.	
Cerulein	Rats	Myrtle leaf extract (100 mg/kg) for 14 days	↓Serum lipase level	(Ozbeyli et al., 2020)
			↓Amylase level	
			↓MDA	
			↑GSH	
			↓MPO	
			↓Pancreatic ROS level	
			↓IL‐1β	
			↓IL‐6	
			↑IL‐10	
			↓Pancreatic edema index	
Croton oil	Rats	Myrtle essential oil (1 and 2 mL/kg) for 10 days	↓MPO activity	(Maxia et al., 2011)
			↓TNF‐α	
			↓IL‐6	
Cypermethrin	Rats	Myrtle leaf extract (1 mL [50 g/L]) for 30 days	↓Blood glucose	(Berroukche et al., 2017)
			↓GPT	
			↑Glutamic oxaloacetic	
			↑Transaminase alkaline phosphatase	
Double ligatures with suture silk	Rats	Myrtle leaf extract (50 mg/kg) for 28 days	↓Plasma total bilirubin	(Sen et al., 2016)
			↓Direct bilirubin	
			↓Alanine aminotransferase	
			↓Aspartate aminotransferase	
			↓Tumor necrosis factor α	
			↓Interleukin‐1β	
Ethanol	Rats	Myrtle berries seed extract (25, 50, and 100 mg/kg) for 2 months	↑Hb	(Jabri et al., 2018)
			↑Ht	
			↓MCV	
			↑MCHC	
			↑Plt	
			↓WBC	
			↓MDA	
			↑SOD	
			↑GPx	
			↑CAT	
			↑GSH	
			↑SH‐groups	
			↓TNF‐α	
			↓IL‐6	
			↓IL‐8	
			↓IL‐1β	
Ethanol/HCl	Rats	Myrtle essential oil (250, 500, and 1000 mg/kg) for 21 days	↓UI	(Mansour et al., 2022)
			↓UP	
			↑GV	
			↑Gastric pH	
			↓NO production	
			↓MDA	
			↑SOD activity	
			↑CAT	
			↑GPx	
Extremely low frequency magnetic fields (ELFMF)	Rats	Myrtle extract (injected 0.5 mg/kg)	↑FRAP values	(Seif et al., 2019)
			↓Plasma POC	
			↓MetHb	
			↓Hemichrome	
Goldblatt's 2K1C	Rats	Myrtle extract (100 mg/kg) for 9 weeks	↓Serum osteopontin levels	(Cevikelli‐Yakut et al., 2020)
			↑IL‐10	
			↓Hippocampal MMP‐13	
			↓CD36 expression	
			↓Neprilysin levels	
			↓AChE activity	
Hot water	Rats	Myrtle ethanol extract (100 mg/kg) twice a day for 48 h	↓MDA	(Ozcan et al., 2020)
			↑GSH	
			↑CAT	
			↑GST	
			↑SOD	
Hot water	Rats	Myrtle leaves extract (100 mg/kg) for 2 days	↑GSH	(Ozcan et al., 2019)
			↓MDA	
			↑SOD	
			↑CAT	
			↑Skin NO levels	
			↑TF activity	
Hydatid cysts protoscoleces	Mice	Essential oil of Myrtle (0.05, 0.1, 0.2, and 0.4 mg/kg) for 14 days	↑Mortality of protoscoleces	(Mahmoudvand et al., 2016)
Iron	Mice	Myrtle leaf extract (50 and 100 mg/kg) for 4 weeks	↓Total serum iron	(Eslami et al., 2018)
			↓Serum Fe^3+^	
			↓Serum AST	
			↓Serum ALT	
			↓Serum ALP	
Liver ischemia–reperfusion	Rats	Myrtle extract	↓AST	(Salouage et al., 2010)
			↓ALT	
			↑MEGX	
Monosodium glutamate and acrylamide	Rats	Myrtle leaf extract (300 mg/kg) for 6 weeks	↑Bcl‐2	(Hassan et al., 2020)
			↓Apoptosis	
			↓PD‐1	
Paracetamol	Rats	Aqueous extract of Myrtle leaves (200 and 400 mg/kg) for 10 days	↓SGPT	(Rupesh et al., 2011)
			↓SGOT	
			↑CAT	
			↓ALP	
			↑SOD	
			↓LPO	
			↓Total bilirubin	
Silver nanoparticles	Mice	Hydrolyzable tannin fraction of *Myrtus communis* (50, 100, and 200 mg/kg) for 90 days	↓Serum AST	(Tavakoli et al., 2020)
			↓Serum ALT	
			↓Serum ALP	
			↑White blood cells	
			↑Red blood cells	
			↑Lymphocytes	
			↑Hb	
Streptozotocin	Rats	Myrtle leaf extract (100 mg/kg) for 4 weeks	↓Blood glucose levels	(Kadıoğlu Yaman et al., 2020)
			↓Ache activities	
			↑Hippocampal CHAT activity	
			↑Neprilysin levels	
			↑α7‐nAChR	
			↑PSA‐NCAM	
			↑BDNF expressions	
Streptozotocin	Rats	Ethanol extract of Myrtle (0.25, 0.5, and 1 g/kg) for 14 days	↓ALT	(Aggul et al., 2022)
			↓AST	
			↓MDA	
			↓Blood glucose levels	
			↑GSH levels	
			↑SOD activities	
Streptozotocin	Rats	Myrtle fruit hydro‐alcoholic extract	↓Serum glucose	(Tas et al., 2018)
			↓Serum lipid	
			↓MDA	
			↑Insulin	
			↑Paraoxonase arylesterase	
			↑SOD	
			↑Whole blood GSH‐Px	
Streptozotocin	Rats	Myrtle extract	↓5‐LOX and 15‐LOX	(El‐Bana et al., 2017)
			↓Lipoxin A4	
			↓TNF‐α	
			↑Insulin	
Surgery	Rats	Myrtle berry seeds extract (25, 50, and 100 mg/kg)	↑Gastric juice pH	(Jabri, Tounsi, et al., 2016)
			↓MDA	
			↑GSH	
			↑SOD	
			↑CAT	
			↑GPx	
			↓Hydrogen peroxide	
			↓Free iron	
			↓Calcium	
Surgery	Rats	Myrtle leaves essential oil (50 mg/kg) for 7 days	↑CAT	(Jabri, Hajaji, et al., 2016)
			↑SOD	
			↑GPx	
			↓Lipid peroxidation	
*Toxoplasma gondii*	Mice	Essential oil of Myrtle (100, 200, and 300 mg/kg/day) for 3 weeks	↓Tissue cyst	(Shaapan et al., 2021)
			↓Diameter of tissue cyst	
			↑IFN‐γ	
			↑IL‐12	

**TABLE 2:** In vitro protective effects of *Myrtus communis* against toxins and noxious agents.

Toxins	Models	Constituents	Results	References
Hydrogen peroxide	K562 cell line	Leaf extract (0.4 mg/mL) for 2 h	↑MDA production	(Ines et al., 2012)
			↑CAT	
			↓GPx1	
			↑GSS	
			↑HMOX2	
			↑TXN	
			↑TXNRD1	
			↑AOE372	
			↑XRCC1	
			↑LIG4	
			↑POLD2	
			↑XRCC5	
			↑GADD45A	
			↑RPA3	
			↑XPA	
			↑hMSH2	
			↑RPA2	
			↑TDG	
			↑PCNA	
			↑DDIT3	
			↑ERCCI	
	HUVEC cell line	Myrtle extract	↑HIF_1α expression	(Raeiszadeh et al., 2018)
			↑VEGF	
			↓COX‐2	
			↓iNOS	
	K562 cell line	Two compound of Myrtle extract	↓Lipid peroxidation	(Hayder et al., 2008)
			↓MDA	
			↑AOE372 expression	
			↑TXN expression	
			↓GPX1 expression	
			↓SEPW1 expression	
			↑XPC expression	
			↑LIG4 expression	
			↑RPA3 expression	
			↑PCNA expression	
			↑DDIT3 expression	
			↓POLD1 expression	
			↓XRCC expression	
			↓MPG expression	
			↓PARP expression	
			↓SHC1 expression	
IL‐1β	BEAS‐2B	Myrtle essential oil	↓IL‐6	(Gülbol Duran & Terzi, 2021)
			↓IL‐10	
			↓NFkB	
LPS	Macrophages	Myrtle essential oil	↓NO production	(Bouzabata et al., 2015)
	KM48 and KM149 cell line	Myrtle essential oil	↓AHL	(Myszka et al., 2020)
			↓PQS	
			↓mRNA level of the *algU* gene	
			↓Exopolysaccharide synthesis	
			↑Expression of the *mucA* gene	
	Hydatid cyst protoscolices	Myrtle extract (5, 50, and 100 mg/mL) for 4 h	↑Activity of caspase 3, 8, and 9	(Shahnazi et al., 2017)
	Human macrophages	Myrtle leaf extract	↓Superoxide production	(Soomro et al., 2019)
			↓NO production	
			↓Hydrogen peroxide production	
			↓NFκB phsphrolation	
	A549 cell line	Myrtle Essential oil (from 31.25 to 200 μg/mL) for 24 h	↑Caspase 3 gene expression	(Bilgic & Duran, 2020)
			↑Caspase 9 gene expression	
			↑P21 gene expression	

### 3.1 Aflatoxin

Aflatoxin, a very poisonous mycotoxin, can withstand freezing and cooking even in extreme temperatures. A variety of aflatoxins are produced by the fungi *Aspergillus flavus*, *Aspergillus nomiu*, and *Aspergillus parasiticus*, which grow in a wide range of temperatures (from 12 to 42°C) (Hosseini et al., 2023; O'Riordan & Wilkinson, 2008; Yahyazadeh et al., 2021). However, the ideal growing temperature range is between 20 and 30°C (Murphy et al., 2006). The fact that aflatoxin can be produced during preharvest and postharvest storage is one of the main problems with the substance. The primary aflatoxin species include types B1, B2, G1, and G2, with B1 being the most dangerous (Ghali et al., 2009). Aflatoxin contamination in humans occurs through the consumption of foods such as eggs, wheat, maize, milk, and dairy products (Mohammadi, 2011). In both acute and chronic situations, it produces nephrotoxicity, hepatotoxicity, immunotoxicity, carcinogenicity, and mutagenicity (Abdel‐Hamid & Firgany, 2015; Barton et al., 2001; Benigni & Bossa, 2011; Meissonnier et al., 2008). In order to produce the trans‐8,9‐dihydro‐8‐(2,6‐diamino‐4‐oxo‐3,4 dihydropyrimid‐5‐ylformamido)‐9‐hydroxy aflatoxin B1 adduct, exo‐8,9‐epoxide, a DNA alkylating agent, is attached to the N7 of guanine (Li, Brown, et al., 2015). This is what gives aflatoxin its carcinogenic properties. Moreover, human cytochrome P450‐3A4.62 partially metabolizes aflatoxin (Bren et al., 2007). Thus, according to the WHO, diseases caused by aflatoxin must be prevented or treated in order to lessen their burden and side effects (Ochieng et al., 2013).

According to the results of their experiment on chicken, Saei et al. concluded that the use of Myrtle essential oil as a treatment reduces the harmful effects of aflatoxin on various aspects, including serum glucose, creatinine (CREA), cholesterol (CHOL), alanine transaminase (ALT), aspartate aminotransferase (AST), alkaline phosphatase (ALP) concentration, feed intake, and body weight gain. Serum AST, ALT, and ALP are highly sensitive markers utilized for diagnosing hepatic damage due to their release into the circulation after cellular damage as cytoplasmic enzymes. A decrease in growth rate is the most significant effect of aflatoxin on poultry. The protective properties of this compound are especially evident in growth performance. These findings suggest that the essential oil of Myrtle may be utilized in chickens to prevent the consequences of aflatoxins in contaminated feed, providing a foundation for further exploration of the relationship between Myrtle essential oil and protection against aflatoxin toxicity. This could potentially improve the health, safety, and quality of poultry products (Saei et al., 2013).

### 3.2 Aluminum

Aluminum is a harmful heavy metal that affects the skeletal, hematopoietic, and respiratory systems (Nayak, 2002). Adenyl cyclase, alkaline phosphatase, and acetylcholinesterase are all inhibited by this metal (Niedworok & Fijałkowski, 2004). As a reactive oxygen species (ROS) producer, aluminum also causes DNA alkylation (El‐Demerdash, 2004).

In a study conducted by Yalman et al., the protective effects of Myrtle against the toxicity caused by aluminum chloride and D‐galactose, which caused Alzheimer's induction in rats, were evaluated. The treatment with Myrtle was found to lower the levels of amyloid beta (Aβ) and 8‐hydroxy‐2‐deoxyguanosine (8‐OHdG), while increasing the levels of neprilysin and superoxide dismutase (SOD). The authors proposed that the protective and therapeutic effects of Myrtle may stem from its phenolic compounds. These results indicate that Myrtle can positively impact cognitive and neuronal functions through its ability to prevent the breakdown of acetylcholine and protect against oxidative stress (Yalman et al., 2022).

### 3.3 Arsenic

Arsenic is widely spread in farmland soil and groundwater, and arsenic concentrations in soil and river water reach the WHO standard limit of 10 μg/L in many areas worldwide (Min et al., 2021; Missimer et al., 2018). Many studies in recent decades have demonstrated arsenic‐induced immunotoxicity, nephrotoxicity, reproductive toxicity, and other negative effects in animals (Hall, 2002; Shao et al., 2018). Over the last few years, evidence has accumulated that excessive arsenic intake can cause a variety of toxic effects, including liver injury, abnormal metabolism, and cardiovascular disease toxicity (Chen et al., 2019; Zhong et al., 2020). Because of the high affinity of the liver, the detoxification organ, for heavy metals, the liver is the main toxicity target of arsenic (Liu et al., 2020).

Naji et al. study the protective effect of Myrtle leaves on decreasing the toxicity of arsenic trichloride in the rat. Myrtle contains abundant quantities of linoleic acid, octane 3,5‐dimethyl, oleic acid, and other compounds. These substances function to combat the harmful effects of toxicants by activating distinct cellular pathways, such as programmed cell death. This results in safeguarding cells from the toxicological properties of heavy metals. Arsenic trichloride increases the PGE_2_ levels and protein activity of the p53 gene, reducing gene expression. The utilization of Myrtle, either on its own or combined with arsenic trichloride, can enhance the levels of PGE and improve the condition (Naji et al., 2018).

### 3.4 Bleomycin

Chemotherapeutic medications are used to treat neoplastic disorders such as testicular carcinoma, ovarian cancer, and malignant pleural effusions. Bleomycin (BLM), a chemotherapeutic drug, is a member of the glycopeptide group derived from the bacteria *Streptomyces verticillus* (Claussen & Long, 1999). This medication is hazardous to healthy organs such as the lungs (Umezawa et al., 1966). BLM therapy has also been related to the dissection of the double strand of DNA in the presence of iron and oxygen via the generation of reactive nitrogen species and ROS (Della Latta et al., 2015).

In an in vivo study, the Myrtle methanolic extract effects on BLM‐induced pulmonary fibrosis in rats were investigated. After treatment with Myrtle leaf extract for 14 days at the dose of 50 mg/kg, lipid peroxidation (LPO) was reduced in BLM‐induced pulmonary fibrosis. The results showed the anti‐inflammatory effects of Myrtle methanolic extract against lung fibrosis. The extract from Myrtle could remarkably inhibit inflammation and fibrosis of the lung parenchyma through preventive and therapeutic methods. This positive effect can be attributed to tissue inflammation reduction and oxidative stress inhibition (Samareh Fekri et al., 2018).

### 3.5 Carbon tetrachloride

Several investigations have found that carbon tetrachloride, a common laboratory reagent and industrial use, causes liver damage (Li, Chen, et al., 2015; Weber et al., 2003). Carbon tetrachloride has been shown to alter lipid profiles, stress oxidative markers, total protein, high‐density lipoprotein (HDL), liver enzymes, inflammatory markers, hepatocytes, and fiber segmentation in the liver (Cogliati et al., 2011; Ebeid et al., 2015). Chronic carbon tetrachloride exposure also causes genotoxicity and DNA fragmentation in rats (Alkreathy et al., 2014).

The use of Myrtle essential oil prior to carbon tetrachloride treatment was able to effectively prevent the rise in hepatic markers and lipid levels in rats. Additionally, this particular substance helped improve biochemical and histological factors compared to the group treated solely with carbon tetrachloride. These findings suggest that Myrtle may contain beneficial compounds capable of combating the harmful effects of carbon tetrachloride intoxication and potentially serving as an effective preventative measure against liver damage. The Myrtle essential oil's contents not only safeguard the plasma membrane's integrity but also enhance the liver's regenerative and reparative ability. These findings imply that the Myrtle essential oil compound has hepatoprotective effects against oxidative stress caused by carbon tetrachloride in rats. This is substantiated by the reduction of thiobarbituric acid reactive substances levels in the liver tissue and liver marker enzymes in the serum (Hsouna et al., 2019).

### 3.6 Carrageenan

Carrageenan‐induced paw edema is a common method for screening anti‐inflammatory activity (Winter et al., 1962). It is a sensitive method for nonsteroidal anti‐inflammatory drugs (NSAIDs) and has long been used to study new NSAIDs (Willoughby & DiRosa, 1972). In addition, carrageenan‐induced inflammation is useful in detecting oral anti‐inflammatory agents (Vinegar et al., 1969). Developing edema induced by carrageenan injection causes an acute and local inflammatory response. In the early phase (0–1 h), histamine, serotonin, and bradykinin are the first mediators elaborated, and prostaglandins and various cytokines such as interleukin‐1β (IL‐1β), interleukin‐6 (IL‐6), interleukin‐10 (IL‐10), and tumor necrosis factor α (TNF‐α) are implicated in the second phase (Crunkhorn & Meacock, 1971).

In a study conducted by Rossi et al. using this model, they found that myrtucommulone has high anti‐inflammatory properties (Maxia et al., 2011). Myrtucommulone is a nonprenylated acyl phloroglucinol contained in Myrtle leaves (Rosa et al., 2003). In their study, this compound was able to reduce the level of inflammatory mediators such as TNF‐α and IL‐1β.

### 3.7 Cerulein

The most well‐studied and commonly used experimental model for acute edematous pancreatitis is cerulein‐induced pancreatitis. Supramaximal pancreatic stimulation with cerulein, cholecystokinin (CCK) analog, causes intra‐acinar activation of trypsinogen in rats (Hofbauer et al., 1998). This decapeptide induces smooth muscles and increases digestive secretions, leading to notable parenchymal fibrosis and pancreatitis criteria (Kim, 2008; Wu et al., 2021).

Ozbeyli et al. studied the effects of Myrtle on acute pancreatitis (AP). Rats were divided into four groups (each one had eight rats): saline‐pretreated control, Myrtle‐pretreated control, saline‐pretreated AP, and Myrtle‐pretreated AP group. Myrtle and saline were administered orally daily (100 mg/kg). After 2 weeks, in cerulein‐induced AP rats, inflammatory factors and cellular and oxidative damage had increased. On the other hand, the administration of Myrtle caused anti‐inflammatory and antioxidant effects, such as a reduction in MDA levels and MPO activity and an elevation in GSH levels. Also, pancreatic ROS release was decreased by Myrtle treatment. The results indicate helpful treatment to protect the pancreas (Ozbeyli et al., 2020).

### 3.8 Cypermethrin

Pesticides, such as insecticides, can create severe harm to organisms even at low concentrations, and long‐term exposure can result in genetic disorders and physiological abnormalities that shorten lifespan (Bernardes et al., 2015; Wojciechowska et al., 2016; Zacharia, 2011). Cypermethrin is a widely used insecticide around the world. Cypermethrin is a broad‐spectrum chemical pyrethroid group insecticide used in agriculture for pest control and crop loss prevention (Seven et al., 2022). Its widespread use has the potential to have a variety of toxic properties on nontarget organisms (Grewal et al., 2010). Cypermethrin is a highly poisonous chemical that can be inhaled, ingested, and absorbed through the skin. Skin irritation, numbness and tingling, itching and burning sensations in the eyes, loss of bladder control, seizures, and eventually death can result from exposure (Sangha et al., 2011; Sharma et al., 2018).

The results of a study by Berroukche et al. showed that the use of the treatment with Myrtle leaves decoction (50 g/L) partially improved the condition of animals. In addition, it reduced the harmful effects of the free radicals produced by cypermethrin toxicity. Furthermore, the application of Myrtle caused a reduction in GPT activity and an elevation in ALP activity (Berroukche et al., 2017).

### 3.9 Ethanol

It is proven that ethanol has a toxic effect on the gastrointestinal system (GIS). It can cause irritation and inflammation in GIS. In addition, chronic consumption of ethanol can cause more serious damage to the GIS, including ulcers, bleeding, and even cancer (Bode & Bode, 1997).

Sumbul et al. studied the anti‐ulcer effect of Myrtle extract on alcohol‐induced, pyloric ligation‐induced, and indomethacin‐induced gastric ulceration in rats. Treatment with Myrtle extract significantly reduced ulcer index and gastric juice volume. Also, it showed an increase in the pH of gastric juice and mucus secretion as compared to the toxic control. The findings indicate that Myrtle can be a potential substance for the treatment of gastric ulcers (Sumbul et al., 2010).

Mansour et al. studied the gastroprotective effect of microencapsulated Myrtle essential oil (MMEO) against acute gastric lesions. Rats were randomly divided into six groups. To induce acute gastric lesions, rats were treated with acidified ethanol solution. They found no mortality in rats after 3 weeks of MMEO administration. In addition, MMEO treatment reduced inflammation and submucosal edema. Treatment with MMEO raised pH levels and volume of gastric juice of stomach and reduced gastric lesions (Mansour et al., 2022).

### 3.10 Extremely low‐frequency magnetic fields (ELFMF)

The International Agency for Research on Cancer has classified ELFMF as a cancer‐causing agent in biological systems. There is evidence that alterations in energy levels can impact the generation and recombination of free radicals (Güler et al., 2012; Timmel et al., 1998).

Seif et al. investigated the protective properties of Myrtle extract against the oxidative effects of ELFMF in adult male rats. The extract improved the erythrocytes' capacity and plasma capacity to handle oxidative circumstances when subjected to ELFMF (Seif et al., 2019).

### 3.11 Paracetamol

Paracetamol, also known as acetaminophen, is a commonly used antipyretic with a history of liver toxicity when blood levels rise above therapeutic levels (Watkins et al., 2006). Toxicity from paracetamol has recently become more difficult to determine due to the increased use of combination medications containing paracetamol, such as over‐the‐counter cold medicine or prescription pain relievers (Dougherty & Klein‐Schwartz, 2012; Graudins, 2014; Kirschner et al., 2016). Paracetamol was discovered to cause liver damage in the original studies of the mechanisms of toxicity by being converted by hepatic cytochrome P450 enzymes to a minor but toxic intermediate metabolite (Jollow et al., 1973; Mitchell, Jollow, Potter, Davis, et al., 1973; Mitchell, Jollow, Potter, Gillette, et al., 1973; Potter et al., 1973).

A study by Pasumarthi Phaneendra et al. investigated the hepatoprotective effects of aqueous extract of Myrtle leaves (200 and 400 mg/kg). The most accurate indicators of liver injury are serum SGPT, SGOT, ALP, and bilirubin. An increase in the activity of these markers in this investigation demonstrated that paracetamol‐induced hepatocellular injury had occurred.

The formulation's efficacy in restoring the damaged liver's normal functional status was demonstrated by a reduction in increased plasma activity of these enzymes that paracetamol‐treated rats developed. The outcomes imply that the extract has a substantial hepatoprotective effect as it prevented paracetamol‐induced lipid peroxidation when administered. The extraction also significantly increased the amounts of antioxidant enzymes and decreased the oxidative damage caused by paracetamol, demonstrating its antioxidant potential. This investigation unequivocally showed that the formulation had strong hepatoprotective effects in preventing paracetamol‐induced liver damage in rats. These effects may be attributable to the formulation's antioxidative and free radical‐scavenging characteristics Rupesh et al. (Rupesh et al., 2011).

### 3.12 Silver nanoparticles

The application of silver nanoparticles in biomedicine is on the rise; however, their potential toxicity has not received adequate attention. The liver acts as an effector against inflammation brought on by silver nanoparticles by imposing changes on the level of functional liver enzymes (AST, ALP, and ALT), which also stimulates anomalies in liver function. To investigate the sub‐chronic toxicity of silver nanoparticles in mice and allay this worry, researchers modified silver nanoparticles with a hydrolyzable tannin fraction from Myrtle. The findings showed that treatment with MC‐AgNPs reduced liver dysfunction in mice (ALT/AST/ALP values), particularly ALP, and the AST enzyme levels were comparable to those in the control group. The study concluded that AgNPs could reduce changes in AST, ALT, and ALP, which may be due to the tannins present in the Myrtle extract as beneficial phytochemical components (Tavakoli et al., 2020).

### 3.13 Streptozotocin

Streptozotocin (STZ) was discovered in a strain of the gram‐positive bacterium *Streptomyces achromogenes* found in soil (Singaram et al., 1979). This medicine is a glucosamine–nitrosourea substance that is especially toxic to the insulin‐producing beta‐cells of the pancreas in mammals; It is used to treat certain cancers of the islets of Langerhans (Rakhshandeh et al., 2022). In medical research, STZ is used to create a diabetes model (Isaev et al., 2018; Szkudelski, 2001). STZ is an alkylating chemical that is widely used to induce hyperglycemia in experimental animals due to its cytotoxicity effect on pancreatic beta cells via DNA damage induction and its stimulation of the inflammatory response (Alkhedaide, 2019).

In a study, Tas et al. investigated the potential health benefits of Myrtle in both normal and diabetic rats. The study found that a hydroalcoholic extract of the fruit caused a significant reduction in blood glucose levels and elevated serum insulin levels in diabetic rats. These may arise because *M. communis* fruit contains biologically active phytochemicals, including flavonoids and other phenolic compounds. This may work by increasing insulin production (enhancing pancreatic function), decreasing insulin resistance (speeding up glucose uptake and metabolism), or decreasing intestinal glucose absorption. These effects were not observed in normal rats. The study suggests that Myrtle may have potential therapeutic applications for diabetes and related conditions (Tas et al., 2018). In another in‐vivo study, the Myrtle effect on ovariectomized diabetes rats was investigated. All rats were divided into five groups. STZ was administered 1 week after ovariectomy to induce diabetes. Administration of Myrtle extract decreased blood glucose levels. It also had positive effects on the cognitive function of rats. These findings suggest that Myrtle could be a treatment for cognitive function pathologies (Kadıoğlu Yaman et al., 2020).

In a 2021 study, the effects of ethanol extract of Myrtle for oxidative stress in STZ‐induced diabetes rats were investigated. Rats were orally administered 3 different doses of ethanol extract of Myrtle berries (0.25, 0.5, and 1 g/kg) for 2 weeks. At the end of the experiment, ALT, AST, MDA, and blood glucose levels of the rats significantly decreased while significant increases in GSH levels and SOD activities were observed (Aggul et al., 2022).

Khodaie and his colleagues investigated the wound healing effect of aqueous extract of Myrtle fruit in diabetes. In this study, 60 rats were divided into six groups. Diabetes was induced in them using streptozotocin. Seven days after disease induction, wounds were created on the dorsolateral surface of rats. Rats were treated with aqueous Myrtle extract and gel containing it for 21 days. Myrtle extract increased the speed of wound healing in diabetic rats. It was also found in histological tests that the plant extract decreases tissue's inflammatory cells and increases collagen and angiogenesis. The results show that the aqueous extract of Myrtle fruit improves the speed of wound healing in diabetes (Khodaie et al., 2021).

### 3.14 Toxoplasma gondii

Toxoplasmosis is one of the most widespread zoonotic parasitic diseases caused by the intracellular parasite *T. gondii* (Mose et al., 2020). Considering the clinical appearance of toxoplasmosis, the disease does not cause any definite signs in healthy people, whereas a severe and even lethal one can be observed in immunocompromised individuals (Elfadaly et al., 2012). In an in vivo study, 48 male mice were divided into control (C) and experimental (Ex) groups. Each group is subdivided into three subgroups. Except for C1, all mice in all groups were infected with *T. gondii* after 3 weeks of treatment. Oral administration of Myrtle essential oil showed a reduction in the number and diameter of tissue cysts.

Furthermore, a study assessed the mRNA levels of various innate immunity mediators, including IFN‐γ and IL‐12, by quantitative real‐time PCR as the immune system, particularly cellular immunity, is one of the most crucial mechanisms for controlling toxoplasmosis. The findings showed that although IFN‐γ and IL‐12 mRNA levels were enhanced in all experimental groups of mice, a substantial rise was observed in mice treated with MCEO (*M. communis* essential oil) at the doses of Ex2 and Ex3 of MCEO when compared to control groups. According to the research, the decrease in parasite load in the infected mice treated with MCEO, which led to the control of *T. gondii* infection, may be related to the improved immune function of the tested mice, particularly their innate immune system (Shaapan et al., 2021).

**FIGURE 3:**
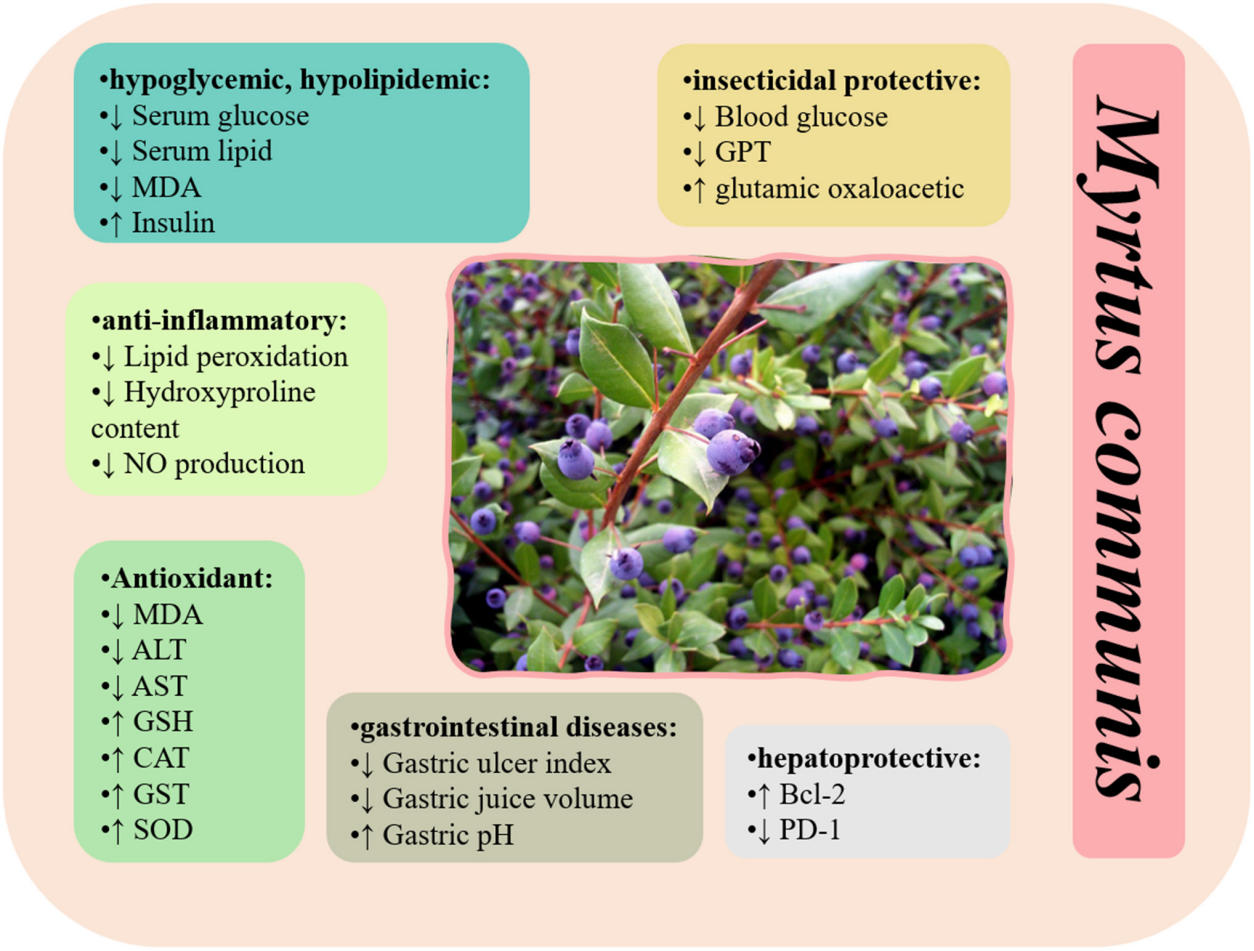
Pharmacological effects of *Myrtus communis*.

The main pharmacological effects of Myrtle are illustrated in Figure 3.

## 4 CONCLUSION

*M. communis*, also known as Myrtle, is a medicinal plant that has been used for centuries for its therapeutic properties. Recent studies have demonstrated its potential as a protective agent against both natural and chemical toxins. In addition, the plant contains several bioactive compounds, such as flavonoids, tannins, and essential oils, which provide a basis for its many health‐promoting effects.

One of Myrtle's most significant protective properties is its high antioxidant content. Studies have shown that the antioxidant properties of Myrtle can protect against harmful substances such as heavy metals, pesticides, and other environmental toxins. Additionally, Myrtle has anti‐inflammatory properties that can help reduce the damage caused by long‐term exposure to toxins. Chemical toxins can cause inflammation, which can lead to organ damage, and this is where the anti‐inflammatory properties of Myrtle can help alleviate the resulting damage. The anti‐inflammatory and antimicrobial properties of Myrtle have also proven effective in alleviating gastrointestinal conditions such as gastric ulcers.

Another significant benefit of the Myrtle plant is its hepatoprotective properties. The liver is a vital organ in the body responsible for filtering toxins and keeping the body healthy. Studies have shown that consuming Myrtle can protect the liver against chemical toxins which can help it function more efficiently and prevent damage. Finally, the multipurpose nature of Myrtle means it can be used to treat a wide range of health conditions caused by exposure to toxins. The plant has been traditionally used to treat several conditions, including respiratory and digestive illnesses, infections, and wounds. In addition, recent studies show that Myrtle may also have a preventive effect against cancer, thanks to its ability to inhibit the growth of tumor cells. In conclusion, Myrtle is a promising natural remedy for treating and preventing various toxicological disorders. However, more research is needed to unlock its full potential and develop targeted therapies.

## AUTHOR CONTRIBUTIONS

Mohammad Mahdi Dabaghi, Mohammad Saleh Fadaei, Hesan Soleimani Roudi, Vafa Baradaran Rahimi, and Vahid Reza Askari wrote the first draft of the manuscript. All authors contributed to writing the project and read and approved the final manuscript submission. This study has been done by the authors mentioned in this article, and the authors will bear all responsibilities related to the contents of this article.

## FUNDING INFORMATION

No funding information provided.

## CONFLICT OF INTEREST STATEMENT

The authors declare no conflict of interest.

## ETHICS STATEMENT

This is a review article. Ethical approval is not required for the study.

## Data Availability

No data were used to support this study.
